# Synergistic inhibition of prostate cancer cell lines by a 19- *nor* hexafluoride vitamin D_3_ analogue and anti-activator protein 1 retinoid

**DOI:** 10.1038/sj.bjc.6690018

**Published:** 1999-09-24

**Authors:** M J Campbell, S Park, M R Uskokovic, M I Dawson, L Jong, H P Koeffler

**Affiliations:** Division of Hematology/Oncology, Cedars-Sinai Medical Center, UCLA School of Medicine, Los Angeles, CA 90048, USA; Hoffman-La Roche, Nutley, NJ 07110, USA; SRI International, Menlo Park, CA 94025, USA

**Keywords:** vitamin D_3_, retinoids, activator protein 1, growth inhibition, prostate cancer

## Abstract

The secosteroid hormones, all- *trans*- and 9- *cis* -retinoic acid and vitamin D_3_, have demonstrated significant capacity to control proliferation in itro of many solid tumour cell lines. Cooperative synergistic effects by these two ligands have been reported, and it is, therefore, possible that greater therapeutic effects could be achieved if these compounds were administered together. The role of retinoid-dependent anti-activator protein 1 (anti-AP-1) effects in controlling cancer cell proliferation appears significant. We have utilized an anti- AP-1 retinoid [2-(4,4-dimethyl-3,4-dihydro-2H-1 benzopyran-6-yl)carbonyl-2-(4-carboxyphenyl)-1,3,-dithiane; SR11238], which does not transactivate through a retinoic acid response element (RARE), and a potent vitamin D_3_analogue [1α,25(OH)_2_-16-ene-23-yne-26,27-F_6_-19-*nor* -D_3_, code name LH] together at low, physiologically safer doses against a panel of prostate cancer cell lines that represent progressively more transformed phenotypes. The LNCaP (least transformed) and PC-3 (intermediately transformed) cell lines were synergistically inhibited in their clonal growth by the combination of LH and SR11238, whereas SR11238 alone was essentially inactive. DU-145 cells (most transformed) were completely insensitive to these analogues. LNCaP cells, but neither PC-3 nor DU-145, underwent apoptosis in the presence of LH and SR11238. Transactivation of the human osteocalcin vitamin D response element (VDRE) by LH was not enhanced in the presence of SR11238, although the expression of E-cadherin in these cells was additively up-regulated in the presence of both compounds. These data suggest the anti-AP-1 retinoid and the vitamin D_3_ analogue may naturally act synergistically to control cell proliferation, a process that is interrupted during transformation, and that this combination may form the basis for treatment of some androgen-independent prostate cancer. © 1999 Cancer Research Campaign


*British Journal of Cancer* (1999) **79**(1), 101–107
© 1999 Cancer Research Campaign

### Synergistic inhibition of prostate cancer cell lines by a 19-*nor* hexafluoride vitamin $D_3$ analogue and anti-activator protein 1 retinoid

**MJ Campbell[1], S Park[1], MR Uskokovic[2], MI Dawson[3], L Jong[3] and HP Koeffler[1]**

[1]Division of Hematology/Oncology, Cedars-Sinai Medical Center, UCLA School of Medicine, Los Angeles, CA 90048, USA; [2]Hoffman-La Roche, Nutley, NJ 07110, USA; [3]SRI International, Menlo Park, CA 94025, USA

**Summary** The secosteroid hormones, all-*trans*- and 9-*cis*-retinoic acid and vitamin $D_3$, have demonstrated significant capacity to control proliferation in vitro of many solid tumour cell lines. Cooperative synergistic effects by these two ligands have been reported, and it is, therefore, possible that greater therapeutic effects could be achieved if these compounds were administered together. The role of retinoid-dependent anti-activator protein 1 (anti-AP-1) effects in controlling cancer cell proliferation appears significant. We have utilized an anti-AP-1 retinoid [2-(4,4-dimethyl-3,4-dihydro-2H-1 benzopyran-6-yl)carbonyl-2-(4-carboxyphenyl)-1,3,-dithiane; SR11238], which does not transactivate through a retinoic acid response element (RARE), and a potent vitamin $D_3$ analogue [1α,25$(OH)_2$-16-ene-23-yne-26,27-$F_6$-19-*nor* -$D_3$, code name LH] together at low, physiologically safer doses against a panel of prostate cancer cell lines that represent progressively more transformed phenotypes. The LNCaP (least transformed) and PC-3 (intermediately transformed) cell lines were synergistically inhibited in their clonal growth by the combination of LH and SR11238, whereas SR11238 alone was essentially inactive. DU-145 cells (most transformed) were completely insensitive to these analogues. LNCaP cells, but neither PC-3 nor DU-145, underwent apoptosis in the presence of LH and SR11238. Transactivation of the human osteocalcin vitamin D response element (VDRE) by LH was not enhanced in the presence of SR11238, although the expression of E-cadherin in these cells was additively up-regulated in the presence of both compounds. These data suggest the anti-AP-1 retinoid and the vitamin $D_3$ analogue may naturally act synergistically to control cell proliferation, a process that is interrupted during transformation, and that this combination may form the basis for treatment of some androgen-independent prostate cancer.

**Keywords:** vitamin $D_3$; retinoids; activator protein 1; growth inhibition; prostate cancer

The use of vitamin $D_3$ analogues in combination with either naturally occurring or synthetic retinoids may form an attractive therapy for androgen-independent metastatic prostate cancer. To investigate this potential, we have examined the effect of a potent vitamin $D_3$ analogue and a synthetic anti-activator protein 1 (anti-AP-1) retinoid on clonal proliferation and investigated the possible mechanism of the observed cooperative effects.

Prostate cancer has become the most frequently diagnosed, non-skin cancer among American men and the second leading cause of cancer mortality among this group (Parker et al, 1997). Despite the increase in the incidence of the disease, no successful long-term therapies exist if the cancer progresses beyond the prostate capsule. Metastatic growth of prostate cancer tissue may be controlled, and even a long-term remission induced, by blockade of endogenous androgen stimulation. Unfortunately, the disease often re-emerges within a few years in a lethal, poorly differentiated and androgen-independent form. The shortage of curative therapies for such a widespread disease has resulted in a large impetus to develop alternatives therapies. Drugs that have been intensively studied in recent years are biological modifiers of growth, either alone or in combinations to retard cell proliferation (Novichenko et al, 1995; Campbell et al, 1998*a*), promote cell death (Danesi et al, 1994; Welsh, 1994; Li et al, 1995; Planchon et al, 1995) and/or induce differentiation of cells to a terminally mature, non-dividing stage (Samid et al, 1993; Liu et al, 1994; Hseih et al, 1995).

Various potential biological modifiers have been investigated, including the physiologically active metabolites of both vitamins A and D (Niles, 1995; Kantarjian et al, 1996) namely all-*trans*-retinoic acid (ATRA) and its isomer 9-*cis*-retinoic acid (9cRA) and 1α,25-dihydroxyvitamin $D_3$ [1α,25$(OH)_2D_3$]. We and others have shown that these compounds, at relatively high doses, can inhibit the in vitro growth of cancer cells from several different tissues, including prostate (Norman et al, 1990; Bollag, 1994; Lotan, 1994; Peehl et al, 1994; Feldman et al, 1995; Saunders et al, 1995; Elstner et al, 1996; Campbell et al, 1997*a*).

One route by which vitamin $D_3$ compounds and retinoids mediate their activity is by binding with specific nuclear receptors; for example 1 α,25$(OH)_2D_3$ interacts exclusively with the vitamin $D_3$ receptor (VDR); ATRA and 9cRA with the retinoic acid receptors (RAR), and 9cRA also with the retinoid X receptor (RXR) (Mangelsdorf et al, 1995). These nuclear receptors are part of the steroid hormone superfamily and act as ligand-inducible transcription factors. They exert specific cellular effects by binding to hexameric response elements within the promoter/enhancer regions and, thereby, regulating transcription of target genes, some

*Received 26 January 1998*
*Revised 1 June 1998*
*Accepted 6 June 1998*

*Correspondence to:* MJ Campbell, Department of Immunology, Medical School, University of Birmingham, Edgbaston, Birmingham B15 2TT, UK

of which are associated with inhibition of proliferation, induction of differentiation or apoptosis. Evidence has also emerged that these compounds work together, for example, the promoter of the cyclin-dependent kinase inhibitor (CDKI) ($p21^{waf1}$), which appears to be important for many of the antiproliferative actions of these compounds, has response elements for both $1\alpha,25(OH)_2D_3$ [vitamin D response element (VDRE)] and ATRA [retinoic acid response element (RARE)] (Liu et al, 1996*a*, 1996*b*). Similarly, we (Elstner et al, 1996) and others (Brown et al, 1994; Blutt et al, 1997) have demonstrated synergistic inhibition and differentiation of various cancer cell lines by these drugs. Separate from the transcriptional activation profile of $1\alpha,25(OH)_2D_3$ and ATRA are other effects that appear to be associated with post-transcriptional regulation of protein expression. For example, $1\alpha,25(OH)_2D_3$ has been shown to increase expression of the CDKI $p27^{kip1}$ in HL-60 leukaemic cells by increasing the rate of mRNA translation and extending protein half-life, but not by altering the mRNA level (Hengst and Reed, 1996; Wang et al, 1996). Similar post-transcriptional effects have been attributed to retinoids in the regulation of another CDKI ($p21^{waf1}$) (Schwaller et al, 1993; Li XS et al, 1996).

Steroid hormone receptors also appear to regulate indirectly other transcriptional pathways via a mechanism known as AP-1 repression. Mitogens, such as cytokines, induce dimerization of the AP-1 proteins, for example c-Jun and c-Fos, into an active form which transactivates genes associated with growth. The potential of this pathway to trigger unregulated growth has been realized in cellular transformation involving Fos and Jun oncoproteins. The thyroid and RAR-α receptors participate in the normal regulation of the AP-1 complex by down-regulating c-*fos* expression (Perez et al, 1993). The relationship between these two opposite regulatory pathways allows for fine-tuning of responses with the possibility of synergistic positive and negative effects (Simonson, 1994; Alroy, 1995; Liu et al, 1995). Both sets of transcriptional elements converge on the regulation of key growth factors, thereby allowing their spatial and temporal expression. For example, interleukin 2 and transforming growth factor $\beta_1$ are positively regulated by AP-1 proteins and negatively controlled by ATRA (Salbert et al, 1993; De Grazi et al, 1994). Correspondingly, deregulation of this process occurs in cancer cells, for example retinoid-resistant breast cancer cell lines (e.g. BT-20) have a high level of endogenous AP-1 activity (Burg et al, 1995), and osteosarcomas may have abnormally high expression of osteocalcin as a result of deregulated AP-1 activity (Jaaskelainen et al, 1994).

Clinical applications of $1\alpha,25(OH)_2D_3$ and ATRA alone at doses that are pharmacologically active have been curtailed because of side-effects. Vitamin $D_3$ is limited to topical application, most notably for the treatment of psoriasis, and although retinoids have achieved remarkable success in the treatment of acute promyelocytic leukaemia (APL) and the prevention of secondary lesions in individuals with head and neck cancers (Huang et al, 1988; Hong et al, 1990), both vitamin analogues have toxicities. For example, many potent vitamin $D_3$ analogues when injected into mice at doses that are active in vitro produce lethal hypercalcaemia (Pakkala et al, 1995). ATRA can produce skin, mucous membrane and hepatic toxicities as well as a high teratogenicity (Morosetti and Koeffler, 1996). Furthermore, over several months of ATRA therapy, APL cells develop insensitivity to the differentiating effects of ATRA. This resistance is multifactorial, which can include abnormal ligand binding and altered transcriptional activation (Rosenauer et al, 1996).

We have previously discovered that the potent analogue of vitamin $D_3$ [$1\alpha,25\text{-}(OH)_2$-16-ene-23-yne-26,27-$F_6$-19*nor*-$D_3$ (code name LH)] inhibits clonal proliferation of LNCaP, PC-3 and DU-145 prostate cancer cells (Campbell et al, 1997*a*). Unfortunately, this analogue also causes hypercalcaemia at high doses (Pakkala et al, 1995). In a preliminary study to enhance the action of this compound, we investigated combinations of LH with various naturally occurring and receptor-selective retinoids that transactivate through either RARE or RXRE (Campbell et al, 1998*b*) and demonstrated that the capacity for synergistic effects was reduced with increased cellular transformation. We have now investigated a synthetic retinoid that has predominately anti-AP-1 effects, as few anti-cancer studies have focused on the contribution of AP-1 repression to retinoid-mediated inhibitory effects. One such retinoid is SR11238, which has approximately 100% of the anti-AP-1 effect of ATRA but has minimal ability to transactivate through a RARE (Fanjul et al, 1994). In this study, we show that combined low doses of LH with SR11238 have prominent antiproliferative effects. We also examined the mechanism of this inhibition by considering various target proteins, such as $p21^{(waf1)}$ and E-cadherin, which we and others have previously shown to be modulated by vitamin $D_3$ and retinoids in cancer cells (Liu et al, 1996; Munker et al, 1996; Campbell et al, 1997*a,b*). Their effects on the induction of apoptosis and transactivation of the vitamin D response element (VDRE) present in the upstream region of the human osteocalcin gene were also examined.

### MATERIALS AND METHODS

### Cells

Human prostate cancer cell lines were obtained from ATCC (Rockville, MD, USA) and maintained as recommended; LNCaP was established from a metastatic lesion in the supraclavicular lymph node of a patient with prostate cancer; PC-3 was derived from a primary adenocarcinoma of the prostate; and DU-145 was established from prostate cancer metastatic to the brain. LNCaP was maintained in RPMI with 10% fetal calf serum (FCS); PC-3 and DU-145 were grown in Dulbecco's modified Eagle medium (DMEM) with 10% FCS.

### Vitamin $D_3$ analogues and retinoids

The vitamin $D_3$ analogue [$1\alpha,25(OH)_2$-16-ene-23-yne-26,27-$F_6$-19-*nor*- $D_3$, code name LH], ATRA, and the synthetic anti-AP-1 retinoid [2-(4,4-dimethyl-3,4-dihydro-2H-1benzopyran-6-yl)-carbonyl-2-(4-carboxyphenyl)-1,3,-dithiane] (SR11238) were kept in stock vials at $10^{-3}$ M in ethanol at –20°C in the dark. For experimental use, these solutions were diluted in normal medium immediately before use.

### Colony formation in soft agar

The antiproliferative potency of LH and retinoids was determined by single- and combination-dose ($10^{-9}$ M) studies in soft agar. Trypsinized and washed single-cell suspensions of LNCaP, PC-3 and DU-145 prostate cancer cells from 80% confluent cultures were enumerated and plated into 24-well, flat-bottomed plates using a two-layer soft agar system with $1 \times 10^3$ cells in 400 μl of media per well, as described previously (Munker et al, 1986). The cells were grown in either RPMI or DMEM. Both layers were prepared with agar (1%) equilibrated at 42°C. Before addition of

© Cancer Research Campaign 1999

the bottom layer to the plate, the LH and either ATRA or SR11238 (final concentration $10^{-9}$ M) were pipetted into the wells and then, after this layer had set, the top layer containing cells was pipetted into the wells. Stock solutions of LH, ATRA and SR11238 and the experimental plates were kept in the dark to minimize UV-catalysed degradation. After 14 days of incubation, the colonies (≥ 50 cells) were counted using an inverted microscope. All experiments were done at least three times in triplicate dishes per experimental point. Dose–response studies with SR11238 were undertaken in the same assay system.

### Measurement of apoptosis

Combinations of LH and SR11238 were investigated for their capacity to induce apoptosis of LNCaP, PC-3 and DU-145 cells. These cells were exposed to either LH or SR11238 alone or a combination of each at $10^{-7}$ M for 4 days, with fresh analogues added at day 2. Total cells, both in the media and those adhering to the plastic, were harvested and fixed in 1% methanol-free formaldehyde for 15 min and washed in phosphate-buffered saline (PBS) (Li and Daryzynkiewcz, 1995). The cell concentration was corrected to $1 \times 10^6$ cells $ml^{-1}$ and fixed in 5 ml of 70% ethanol. Single- and double-strand DNA breaks were labelled with bromodeoxyuridine triphosphate (BrD-UTP) for 40 min at 37°C with terminal transferase (Boehringer Mannheim, Indianapolis, IN, USA). The cells were permeabilized with a 0.3% solution of Triton-X 100 in 0.5% bovine serum albumin (BSA) in PBS, DNA breaks were tagged by the incorporation of BrDU and then identified using a fluorescein isothiocyanate (FITC) conjugated anti-BrDU antibody. Cells were stained with propidium iodide (PI) for 30 min, and green fluorescence was measured by FACS analysis at 510–550 nm.

### Transactivation assays

PC-3 cells ($1 \times 10^7$) were plated in normal media in culture dishes to give 70% confluent cultures. The cells were washed and maintained in DMEM with 10% charcoal-stripped fetal calf serum (CS-FCS) for 24 h before transfection, and then transfected overnight with 3 μg of pBLCAT2 plasmid with the VDRE-containing region of the human osteocalcin promoter, as described by Kuno et al (1994), and 3 μg of cytomegalovirus (CMV) promoter luciferase plasmid (internal standard) in the presence of 18 μg of lipofectamine (Gibco, Grand Island, NY, USA). After transfection, the cells were trypsinized, washed and divided into groups which were cultured in DMEM with 10% CS-FCS with either LH or SR11238 separately, each at $1 \times 10^{-7}$ M, or together at a combined concentration of $1 \times 10^{-7}$ M ($5 \times 10^{-8}$ M each). Treated and non-treated cultures were harvested after 48 h for chloramphenicol acetyltransferase (CAT) reporter assays. After measurement of light production from luciferase and normalization of lysate volume, CAT assays were performed as described previously (Rosenthal, 1987). Relative intensities of acetylated and non-acetylated chloramphenicol were measured on an AMBIS 4000 image analyser (Ambis, San Diego, CA, USA), and the percentage of conversion was calculated. Increases in CAT activity were compared with reporter gene activities in the absence of either retinoid or vitamin $D_3$ analogue. As controls for determination of the linear region of CAT activity, two standards were used. As a negative control, pBLCAT2 plasmid without the human osteocalcin VDRE promotor region was used; and, as a positive control, a CAT plasmid containing a CMV promoter region was used.

### Analysis of protein expression

The effects of LH and SR11238 on the expression of p21(*waf l*) and E-cadherin were examined. We previously found that significant increases in expression of p21(*waf l*) and E-cadherin occurred after 96 h exposure of LNCaP cells to LH (Campbell et al, 1997*b*). We, therefore, examined the effects on subconfluent cultures of LNCaP when exposed to LH and SR11238, either alone (at $1 \times 10^{-7}$ M each) or in combination (at $5 \times 10^{-8}$ M each) for 4 days. Lysates were compared with non-treated cells by sodium dodecyl sulphate polyacrylamide gel electrophoresis (SDS-PAGE) as previously described (Zhang et al, 1995). Briefly, extracts from $1.5 \times 10^6$ cells were boiled in sample buffer for 5 min and loaded onto a 12.5% (p21(*waf l*)) or 7.5% (E-cadherin) SDS-polyacrylamide gel. After electrophoresis at 150 V, the proteins were transferred to Immobilon-P membrane (Millipore, Bedford, MA, USA), blocked with Tris-buffered saline Tween 20 (0.1%) (pH 7.5), 1% gelatin for 1 h, then incubated with antibodies to either p21(*waf l*) (Oncogene Research Products, Cambridge, MA, USA) or E-cadherin (Transduction Laboratories, Lexington, KY, USA). The proteins were then detected using an enhanced chemiluminescence (ECL) system (Amersham Life Sciences, Little Chalfont, Bucks, UK). Comparison of non-specific, background, protein bands and staining of the membrane with Ponceau S were used to adjust the level of lysate added to ensure even loading of protein. Densitometry was performed on bands to quantify the changes in the expression of target proteins.

### Statistical analysis

The interactions of two compounds were assessed by measuring the mean effect of either LH or retinoid acting alone ( ± s.e.m.). The combination of the mean clonal inhibition for each compound acting alone was the predicted combined effect. The mean observed combined clonal inhibition was then compared with this value using the Student's *t*-test. Classification of the inhibitory effects were as follows: synergistic effects were those with an experimental value significantly greater than the predicted value, additive effects were those in which the experimental value did not significantly differ from the predicted value, subadditive effects were those in which the experimental value was significantly less than the predicated value and 'squelching' effects were those in which the experimental value was significantly lower than either agent acting alone.

Other statistical analyses were performed using the Student's *t*-test.

### RESULTS

### Effects of vitamin $D_3$ analogue LH, ATRA and anti-AP-1 retinoid SR11238 on clonal proliferation of human prostate cancer cells

Figure 1 shows the effects of these compounds either alone or in combination at the relatively low dose of $10^{-9}$ M on the clonal growth of prostate cancer cells. LH ($10^{-9}$ M) inhibited 24 ± 2.6% (mean ± s.e.) clonal growth of LNCaP cells, and ATRA and SR11238 ($10^{-9}$ M) alone resulted in a mean colony inhibition of 12 ± 5% and 11 ± 3.6% respectively. Together, LH and either ATRA or SR11238 ($10^{-9}$ M) synergistically inhibited growth by a

© Cancer Research Campaign 1999 

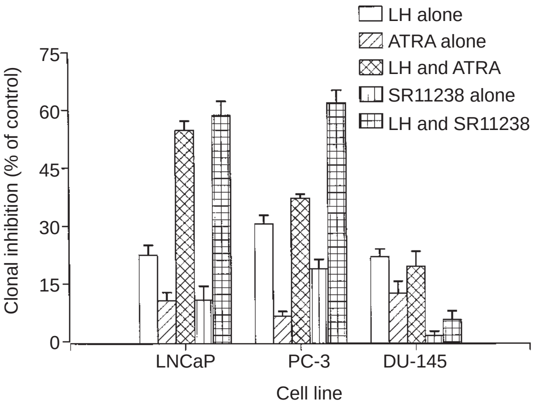

**Figure 1** Inhibition of clonal growth of prostate cancer cells exposed to LH, ATRA and SR11238 alone or in combination ($10^{-9}$ M). Results expressed as per cent (mean ± s.e.m.) of non-treated control cells. Each point represents the mean of at least three experiments with triplicate dishes. Cells were treated with analogue LH, ATRA and SR11238 either separately at $10^{-9}$ M each or together at $5 \times 10^{-9}$ M each (total analogue concentration $10^{-9}$ M)

**Table 1** Summary of clonal inhibition by the combinations of LH and various retinoids

| Combination | Clonal inhibition LNCaP | PC-3 | DU-145 |
|---|---|---|---|
| ATRA + LH[a] | Syn* | Add | Sque*** |
| SR11238 + LH | Syn* | Syn** | Sque* |

[a]The interactions of two compounds were assessed by measuring the means of either LH or retinoid acting alone (± s.e.m.). The combination of the mean clonal inhibition for each compound acting alone was the predicted combined effect. The mean observed combined clonal inhibition was then compared with this value using the Student's *t*-test. Classification of the inhibitory effects were as follows: synergistic effects were those with an experimental value significantly greater than the predicted value, additive effects were those in which the experimental value did not significantly differ from the predicted value, subadditive effects were those in which the experimental value was significantly less than the predicted value and squelching effects were those in which the experimental value was significantly lower than either agent acting alone. Sque, squelching effects; Add, additive effects; Syn, synergistic effects. *$P < 0.001$; **$P < 0.05$; ***$P < 0.01$.

mean 56 ± 4% ($P < 0.05$) and 59 ± 3.6% ($P < 0.001$) compared with the non-treated control.

The PC-3 cells were less sensitive than LNCaP cells to the combined effects of LH plus ATRA (mean inhibition was 38% ± 1.5%). However, the combination of LH plus SR11238 retained significant potency resulting in a synergistic inhibition of clonal growth (62 ± 3.2%) ($P < 0.05$). In contrast, DU-145 cells were insensitive to both of these combinations and, indeed, displayed squelching effects with either combination. These results are summarized in Table 1.

Separate dose–response studies of SR11238 alone revealed this compound to be inactive against all three cell lines with less than 30% colony inhibition even at $10^{-6}$ M (data not shown).

### Induction of apoptosis

Of the three prostate cancer cell lines, only LNCaP cells underwent significant apoptosis in the presence of either LH or SR11238. LNCaP cells underwent a small, but constant, level of apoptosis under normal growth conditions (approximately 3%). In the presence of LH, a small but statistically significant ($P < 0.01$) increase in apoptosis occurred (mean 9.4 ± 0.6%). SR11238 alone also significantly ($P < 0.01$) increased the adoptotic cells (11.5 ± 1.5%). The combination of LH and SR11238 did not significantly differ from the predicted combined value and was considered an additive effect.

### Transduction through a VDRE

The human osteocalcin promoter contains a well-characterized VDRE, which has a RXR and VDR half-sites, and a closely associated AP-1 site. Studies have suggested that the *Jun/Fos* AP-1 complex is able to inhibit VDR binding to the VDRE, thereby down-regulating gene expression (Jaaskelainen et al, 1994). Therefore, we hypothesized that the synergistic inhibition by LH and SR11238 may be due to the reduction in AP-1's antagonism to transactivation of the VDRE by VDR. Transient transfection assays in PC-3 cells showed that LH and SR11238 alone ($10^{-7}$ M) resulted in a mean increase in CAT activity of 3.5-fold ± 0.25-fold and 0.5-fold ± 0.06-fold respectively; whereas, the combination of the two compounds did not significantly alter the CAT activity from that of LH alone (mean 3.6-fold ± 0.12-fold increase over control cells not exposed to ligand) (Figure 2).

Transactivation through VDRE-containing plasmid, in the absence of ligand was not significantly greater than that achieved through a minimal promoter-containing plasmid that lacked a VDRE (data not shown).

### p21$^{(wafl)}$ and E-cadherin protein expression

Analysis of the p21$^{(wafl)}$ protein expression revealed that only LH but not SR11238 modulated it, and the combination of both compounds was not greater than LH alone (data not shown). E-cadherin expression was enhanced by both LH and SR11238

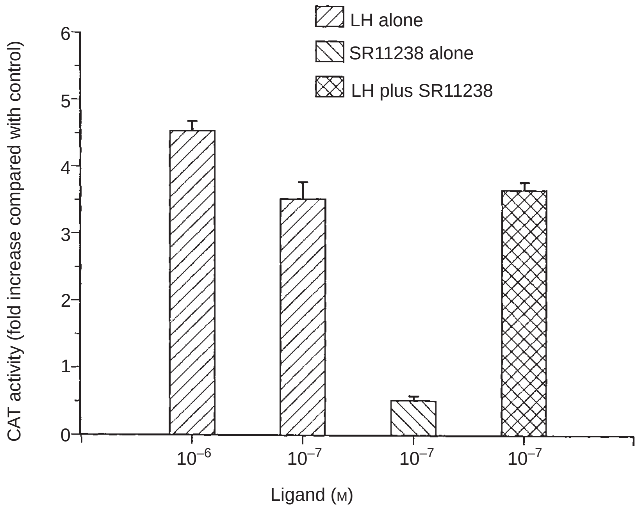

**Figure 2** Transactivation of a VDRE-CAT reporter construct. PC-3 cells transiently transfected with the human osteocalcin CAT reporter construct were exposed separately to analogue LH ($10^{-6}$ or $10^{-7}$ M) and SR11238 ($10^{-7}$ M) or together at $5 \times 10^{-8}$ M each (total analogue concentration $10^{-7}$ M). CAT activity was measured as described in the Materials and methods section

© Cancer Research Campaign 1999

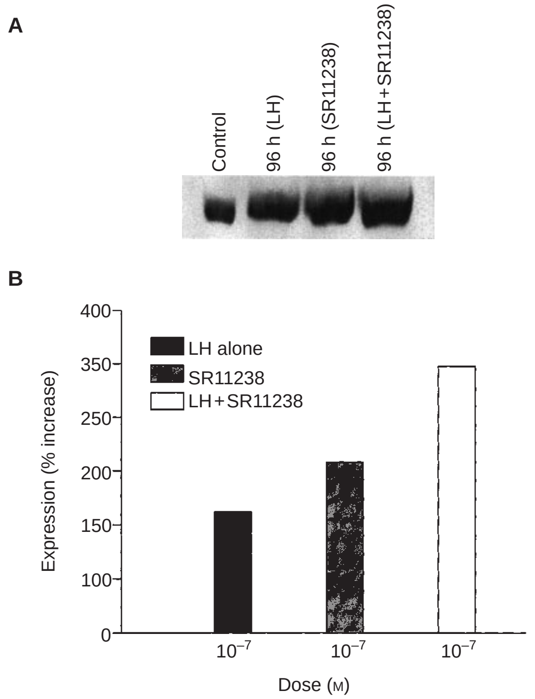

**Figure 3** Modulation of E-cadherin by LH and/or SR11238. Expression of E-cadherin by LNCaP cells was compared with untreated control after treatment with analogue LH and SR11238 either separately at $10^{-7}$ M each, or together at $5 \times 10^{-8}$ M each (total analogue concentration $10^{-7}$ M). (**A**) Cell lysates were resolved by SDS-PAGE, and E-cadherin was detected by Western blot analysis using the antibody described in Materials and methods. (**B**) Densitometry was performed on the bands and expressed as a percentage increase compared with non-treated control separately at $10^{-7}$ M (210% and 250% increased expression respectively, compared with untreated control). The combination of both together at a total reagent concentration of $10^{-7}$ M (each at $5 \times 10^{-8}$ M) produced a greater increase (350% increased levels compared with untreated controls) (Figure 3).

### DISCUSSION

In the present study, we attempted to enhance the pharmaceutical index of the potent vitamin $D_3$ analogue LH by combination treatment with a conformationally restricted retinoid SR11238 that has been reported to have the AP-1 repressive capacities of ATRA, but not its ability to transactivate through the RARE (Fanjul et al, 1994). One of the more interesting findings of the present study was the observation that LH plus SR11238 produced a dramatic, synergistic inhibition of the LNCaP and PC-3 cells. Previously, we have shown LH to be a potent growth inhibitor of prostate cancer cells (Campbell et al, 1997*a*). In the current study, we found that, although the retinoid SR11238 alone had very low inhibitory activity, its combination with LH caused synergistic inhibition of proliferation. A similar finding was reported in a recent study which showed that anti-AP-1 retinoids could be as potent as ATRA at inhibiting transformation of murine JB6 cell (Li JJ et al, 1996). The current study is of special note because the LNCaP and PC-3 cells were insensitive to SR11238 alone, but were significantly inhibited by SR11238 in combination with LH.

AP-1 repression has been investigated intensively and the inhibition of *Jun/Fos*-mediated gene transcription by steroid hormone receptors has been demonstrated in several cell types (Simonson, 1994; Alroy, 1995; Liu et al, 1995). Similarly, synthetic anti-AP-1 retinoids and vitamin $D_3$ analogues have been shown to repress synergistically the TPA-induced AP-1 activity in MCF-7 breast cancer cells (Chen et al, 1995). We explored how LH and SR11238 might be mediating their synergistic clonal growth inhibition of LNCaP and PC-3 cells. To investigate this, we utilized a human osteocalcin promoter with the VDRE and AP-1 sites in close proximity attached to the CAT reporter gene. We hypothesized that if LH and SR11238 acted synergistically to enhance VDRE-mediated growth inhibition, we may observe a corresponding synergistic increase in VDRE-mediated CAT activity. However, the LH and SR11238 combination did not increase transactivation of the VDRE-containing reporter. Similarly, we did not observe a synergistic up-regulation, in the presence of both compounds, of p21$^{(waf1)}$ expression, a protein whose gene has multiple VDRE sites within its promoter region. Therefore, although the effects of LH and SR11238 converge to inhibit clonal proliferation, these compounds probably do not affect the same genomic targets. Thus, we do not know, as yet, the mechanism by which SR11238 potentiated the effect of LH.

The expression of E-cadherin was additively up-regulated in the presence of LH and SR11238. This observation is of interest because the loss or down-regulation of E-cadherin protein is observed in many metastatic cancers of epithelial origin. In prostate cancer, 50% of metastases have either low or no expression of E-cadherin (Umbas et al, 1992; Cheng et al, 1996); whereas normal, differentiated prostate cells display high levels of this protein. It has been suggested that the E-cadherin gene may play a role in preventing metastasis and behave as a tumour suppressor (Umbas et al, 1994). Taken together, E-cadherin is a good marker of functional differentiation (Otto et al, 1993; Campbell et al, 1997*a,b*). In the current study, up-regulation of E-cadherin may involve more than exclusively VDRE-mediated pathways because SR11238 did not appear to affect a VDRE reporter construct. Vitamin $D_3$ has been shown to play a physiological role with testosterone to regulate normal prostate cells (Konety et al, 1996). Perhaps retinoids, acting in an anti-AP-1 capacity, also have a physiological role in the control of growth of normal prostate cells.

The extent to which the anti-tumorigenic effects of ATRA are mediated by an anti-AP-1 pathway is undetermined. Previously, we have shown synergistic inhibition of prostate cancer cell proliferation by LH and certain novel and naturally occurring retinoids, and that a loss of synergistic inhibition correlated with loss of expression of RAR-β (Campbell et al, 1998*b*). However, this previous study was not able to assess to what extent the anti-AP-1 effects of retinoids contributed to the inhibitory behaviour. We have now shown, using a similar experimental design, that the contribution of these retinoid effects appears to be highly significant because the effect of LH plus SR11238 was potent in LNCaP and PC-3 cells, whereas that of LH and ATRA was only synergistically inhibitory only in LNCaP cells. This is intriguing because PC-3 cells are more transformed than LNCaP cells (Carrol et al, 1993; Gaddipati et al, 1994; Issacs et al, 1994; Ewing et al, 1995; Tamimi et al, 1996) and, in particular, do not express RAR-β, whereas LNCaP cells do. This is possibly reflected by the reduced sensitivity of PC-3 cells to LH and various retinoids as found in the present study, and more comprehensively in the previous one (Campbell et al, 1998*b*). Strikingly, in PC-3 cells, antiproliferative potency appears to be retained by anti-AP-1

retinoids in combination with LH. This finding suggests that anti-AP-1 effects may contribute significantly to the anti-cancer effects of certain retinoids and may be retained by androgen-independent prostate cancer cells.

In summary, we have demonstrated a significant enhancement of the anti-proliferative effects against androgen-sensitive (LNCaP) and androgen-insensitive (PC-3) prostate cancer cells by the combination, at low and more physiological doses, of a potent vitamin $D_3$ analogue and a synthetic anti-AP-1 retinoid. The mechanism of inhibition did not appear to be increased by up-regulation of purely VDRE-mediated events, but included other cellular events that may represent a convergence between several signalling pathways, including, for example, increased expression of E-cadherin.

### ACKNOWLEDGEMENTS

This research was supported by NIH grants CA43277, CA42710, CA70675-01 and CA26038, a United States Army Grant for Breast Cancer Research, and also in part by the Concern Foundation and the Parker Hughes Trust. Dr H Phillip Koeffler is a member of the UCLA Jonsson Comprehensive Cancer Center and holds an endowed Mark Goodson Chair of Oncology Research at Cedars-Sinai Medical Center, UCLA School of Medicine.

### REFERENCES

Alroy I, Towers TL and Freedman LP (1995) Transcriptional repression of the interleukin-2 gene by vitamin $D_3$: direct inhibition of the NFATp/AP-1 complex formation by a nuclear hormone receptor. *Mol Cell Biol* **15**: 5789–5799

Blutt SE, Allegretto EA, Wesley Pike J and Wiegel NL (1997) 1,25-Dihydroxyvitamin $D_3$ and 9-*cis*-retinoic acid act synergistically to inhibit the growth of LNCaP prostate cells and cause accumulation of cell in $G_1$. *Endocrinology* **138**: 1491–1497

Bollag W (1994) Experimental basis of cancer combination chemotherapy with retinoids, cytokines, 1,25-dihydroxyvitamin $D_3$ and analogs. *J Cell Biochem* **56**: 427–435

Brown G, Bunce CM, Rowlands DC and Williams GR (1994) All-trans retinoic acid and 1α,25-dihydroxyvitamin $D_3$ co-operate to promote differentiation of the human promyeloid leukemia cell line HL60 to monocytes. *Leukemia* **8**: 806–815

Burg B, Slager-Davidov R, Leede B-J, de Laat SW and van der Saag PT (1995) Differential regulation of AP1 activity by retinoic acid in hormone-dependent and -independent breast cancer cell lines. *Mol Cell Endocrinol* **112**: 143–152

Campbell MJ, Elstner E, Holden S, Uskokovic M and Koeffler HP (1997*a*) Inhibition of prostate cancer cell proliferation by 19-nor-hexafluoride vitamin $D_3$ analogs involves induction of $p21^{(wafl)}$, $p27^{(kipl)}$ and E-cadherin. *J Mol Endocrinol* **19**: 15–27

Campbell MJ, Reddy SG and Koeffler HP (1997*b*) Potent vitamin $D_3$ analogs and their 24-oxo metabolites display equally potent inhibition of proliferation against a variety of cancer cell types but do not have the same molecular effects. *J Cell Biochem* **66**: 413–425

Campbell MJ, Dawson M and Koeffler HP (1998*a*) Growth inhibition of DU-145 cancer cells by *Bcl-2* antisense oligonucleotides is enhanced by *N*-(2-hydroxyphenyl) all-*trans* retinamide. *Br J Cancer* **77**: 739–744

Campbell MJ, Park S, Uskokovic M, Dawson MI and Koeffler HP (1998*b*) Expression of RARβ sensitizes prostate cancer cells to growth inhibition mediated by combinations of retinoids and a vitamin $D_3$ analog. *Endocrinology* **139**: 1972–1980

Carrol AG, Voeller HJ, Sugars L and Gelmann EP (1993) p53 oncogene mutations in three prostate cancer cell lines. *Prostate* **23**: 123–134

Chen JY, Penco S, Ostrowski J, Balaguer P, Pons M, Starrett JE, Reczek P, Chambon P and Gronemeyer H (1995) RAR-specific agonist/antagonists which dissociate transactivation and AP1 transrepression inhibit anchorage-independent cell proliferation. *EMBO J* **14**: 1187–1197

Cheng L, Nagabhushan M, Pretlow TP, Amini SB and Pretlow TG (1996) Expression of E-cadherin in primary and metastatic prostate. *Modern Pathol* **148**: 1375–1380

Danesi R, Figg WD, Reed E and Myers CE (1994) Paclitaxel (taxol) inhibits protein isoprenylation and induces apoptosis in PC-3 human prostate cancer cells. *Mol Pharmacol* **47**: 1106–1111

De Grazi U, Felli MP, Vacca A, Farina AR, Maroder M, Cappabianca L, Meco D, Farina M, Screpanti I, Frati L and Gulino A (1994) Positive and negative regulation of the composite octamer motif of the interleukin 2 enhancer by AP-1, OCT-2 and retinoic acid receptor. *J Exp Med* **180**: 1485–1497

Elstner E, Linker-Israeli M, Le J, Grillier I, Said J, Shintaku P, Krajewski S, Reed JC, Binderup L and Koeffler HP (1996) Combination of potent 20-epi-vitamin $D_3$ analog (KH1060) and 9-*cis* retinoic acid inhibits irreversibly clonal growth, decreases bcl-2 expression and induces apoptosis in HL-60 leukemic cells. *Cancer Res* **56**: 3570–3576

Ewing CM, Ru N, Morton RA, Robinson JC, Wheelock MJ, Johnson KR, Barrett JC and Isaacs WB (1995) Chromosone 5 suppresses tumorigenicity of PC3 prostate cancer cells: correlation with re-expression of α-catenin and restoration of E-cadherin function. *Cancer Res* **55**: 4813–4817

Fanjul A, Dawson MI, Hobbs PD, Jong L, Cameron JF, Harlev E, Graupner G, Lu XP and Pfahl M (1994) A new class of retinoids with selective inhibition of AP-1 inhibits proliferation. *Nature* **372**: 107–111

Feldman D, Skowronski RJ and Peehl DM (1995) Vitamin D and prostate cancer. *Adv Exp Med Biol* **375**: 53–63

Gaddipati JP, Mcleod DG, Heidenberg HB, Sesterhenn IA, Finger MJ, Moul JW and Srivastva S (1994) Frequent detection of codon 877 mutation in the androgen receptor gene in advanced prostate cancers. *Cancer Res* **54**: 2861–2864

Hengst L and Reed S (1996) Translational control of p27 accumulation during the cell cycle. *Science* **271**: 1861–1863

Hong WK, Lippman S, Itri LM, Karp DD, Lee JS, Byers R, Schantz SP, Kramer R, Lotan R, Peters LJ, Dimery IW, Brown BW and Goepfert H (1990) Prevention of second primary tumors with isotretinoin in squamous-cell carcinoma of the head and neck. *N Eng J Med* **323**: 795–801

Hsieh TC, Xu W and Chiao JW (1995) Growth regulation and cellular changes during differentiation of human prostatic cancer LNCaP cells as induced by T lymphocyte-conditioned medium. *Exp Cell Res* **218**: 137–143

Huang M, Ye Y, Chen SR, Chai JR, Lu JX, Zhao L, Gu LJ and Wang ZY (1988) Use of all-*trans* retinoic acid in the treatment of acute promyelocytic leukemia. *Blood* **72**: 567–572

Issacs WB, Bova GS, Morton RA, Bussemakers MJ, Brooks JD and Ewing CM (1994) Genetic alterations in prostate cancer. *Cold Spring Harbor Symp Quant Biol* **59**: 653–659

Jaaskelainen T, Pirskanen A, Ryhanen S, Palvima JJ, Deluca, HF and Maenpaa PH (1994) Functional interference between AP-1 and the vitamin D receptor on osteocalcin gene expression in human osteocsarcoma cells. *Eur J Biochem* **224**: 11–20

Kantarjian HM, Estey EH and Keating MA (1996) New chemotherapeutic agents in acute myeloid leukemia. *Leukemia* **10** (suppl. 1): S4–6

Konety BR, Schwartz GG, Acierno JS, Becich MJ and Getzenberg RH (1996) The role of vitamin D in normal prostate growth and differentiation. *Cell Growth Differ* **7**: 1563–1570

Li CJ, Wang C and Pardee AB (1995) Induction of apoptosis by beta-lapachone in human prostate cancer cells. *Cancer Res* **55**: 3712–3715

Li JJ, Dong Z, Dawson MI and Coldurn NH (1996) Inhibition of tumor-promoter-induced transformation by retinoids that transpress Ap-1 without transactivation retinoic acid response element. *Cancer Res* **56**: 481–489

Li X and Daryzynkiewicz Z (1995) Labelling DNA strand breaks with BrdUTP. Detection of apoptosis and cell proliferation. *Cell Prolif* **28**: 571–579

Li XS, Rishi AK, Shao ZM, Dawson MI, Jong L, Shroot B, Reichert U, Ordonez J and Fontana JA (1996) Posttranscriptional regulation of p21 WAF/CIP1 expression in human breast carcinoma cells. *Cancer Res* **56**: 5055–5062

Liu L, Shack S, Stetler-Stevenson WG, Hudgins WR and Samid D (1994) Differentiation of cultured human melanoma cells induced by the aromatic fatty acids phenylacetate and phenylbutyrate. *J Invest Dermatol* **103**: 335–340

Liu M, Lee M, Cohen M, Bommakanti M and Freedman L (1996*a*) Transcriptional activation of the Cdk inhibitor p21 by vitamin D leads to the induced differentiation of the myelomonocytic cell line U-937. *Genes Dev* **10**: 142–153

Liu M, Iavarone A and Freedman LP (1996*b*) Transcriptional activation of the human $p21^{wafl\ cipl}$ gene by retinoic acid receptor. *J Biol Chem* **271**: 31713–31728

Liu W, Hillman AG and Harmon JM (1995) Hormone-independent repression of AP-1-inducible collagenase promoter activity by glucocorticoid receptors. *Mol Cell Biol* **15**: 1005–1013

Lotan R (1994) Suppression of squamous cell carcinoma growth and differentiation by retinoids. *Cancer Res* **54** (suppl.): 1987–1990

Mangelsdorf DJ, Thummel C, Beato M, Herrlich P, Schutz G, Umesono K, Blumberg B, Kastner P, Mark M, Chambon P and Evans RM (1995) The nuclear receptor superfamily: the second decade. *Cell* **83**: 835–839

© Cancer Research Campaign 1999

Morosetti R and Koeffller HP (1996) Differentiation therapy in myelodysplastic syndromes. *Semin Hematol* **33**: 236–246
Munker R, Norman A and Koeffler HP (1986) Vitamin D compounds. Effect on clonal proliferation and differentiation of human myeloid cells. *J Clin Invest* **78**: 424–430
Munker R, Kobayashi T, Elstner E, Norman AW, Uskokovic M, Azhang W, Andreef M and Koeffler HP (1996) A new series of Vitamin D analogs is highly active for clonal inhibition, differentiation, and induction of WAF1 in myeloid leukemia. *Blood* **8**: 2201–2209
Niles RM (1995) Use of vitamins A and D in chemoprevention and therapy of cancer: control of nuclear receptor expression and function. Vitamins, cancer and receptors. *Adv Exp Med Biol* **375**: 53–63
Norman AW, Zhou JW, Henry JM, Uskokovic MR and Koeffler HP (1990) Structure–function studies on analogues of 1 alpha,25-dihydroxyvitamin $D_3$: differential effects on leukemic cell growth, differentiation, and intestinal calcium absorption. *Cancer Res* **50**: 6857–6864
Novichenko N, Konno S, Nakajima Y, Hsieh TC, Xu W, Turo K, Ahmed T and Chiao JW (1995) Growth attenuation in a human prostate cell line mediated by a phorbolester. *Proc Soc Exp Biol Med* **209**: 152–156
Otto T, Rembrink K, Goepel M, Meyer-Schwickerath M and Rubben H (1993) E-cadherin: a marker for differentiation and invasiveness in prostatic carcinoma. *Urol Res* **21**: 359–362
Pakkala S, de Vos S, Elstner E, Rude RK, Uskokovic M, Binderup L and Koeffler HP (1995) Vitamin $D_3$ analogues: effect on leukemic clonal growth and differentiation, and on serum calcium levels. *Leukemia Res* **19**: 65–72
Parker SL, Tong T, Bolden S and Wingo PA (1997) Cancer statistics. *CA Cancer J Clin* **47**: 5–28
Peehl DM, Skowronski RJ, Leung GK, Wong ST, Stamey TA and Feldman D (1994) Antiproliferative effects of 1,25-dihydroxyvitamin $D_3$ on primary cultures of human prostatic cells. *Cancer Res* **54**: 805–810
Perez P, Schönthal A and Aranda A (1993) Repression of c-fos gene expression by thyroid hormone and retinoic acid receptors. *J Biol Chem* **268**: 23538–23543
Planchon SM, Wuerzberger S, Frydman B, Witiak DT, Hutson P, Church D, Wilding G and Boothman A (1995) Beta-lapachone-mediated apoptosis in human promyelocytic leukemia (HL-60) and human prostate cancer cells: a p53-independent response. *Cancer Res* **55**: 3706–3711
Rosenauer A, Raelson JV, Nervi C, Eydoux P, DeBlasio A and Miller Jr WH (1996) Alterations in expression, binding to ligand and DNA, and transcriptional activity of rearranged and wild-type retinoid receptors in retinoid-resistant acute promyelocytic leukemia cell lines. *Blood* **88**: 2671–2682
Rosenthal N (1987) Identification of regulatory elements of clones genes with functional assays. *Methods Enzymol* **152**: 704–720
Salbert G, Fanjul A, Piedrafita FJ, Lu XP, Kim S-J, Tran P and Pfahl M (1993) Retinoic acid receptors and retinoid X receptor-α down-regulate the transforming growth factor-β1 promoter by antagonizing AP-1 activity. *Mol Endocrinol* **7**: 1347–1356
Samid D, Shack S and Myers CE (1993) Selective growth arrest and phenotypic reversion of prostate cancer cells in vitro by nontoxic pharmacological concentrations of phenylacetate. *J Clin Invest* **91**: 2288–2295
Saunders DE, Christensen C, Williams JR, Wappler NL, Lawrence WD, Malone JM, Malviya VK and Deppe G (1995) Inhibition of breast and ovarian carcinoma cell growth by 1,25-dihydroxyvitamin $D_3$ combined with retinoic acid or dexamethasone. *Anti-Cancer Drugs* **6**: 562–569
Schwaller J, Koeffler HP, Nicklaus G, Loetscher P, Nagel S, Fey MF and Tobler A (1993) Posttranscriptional stabilization underlies p53-independent induction of p21 in differentiating human leukemic cells. *J Clin Invest* **95**: 973–979
Simonson MS (1994) Anti-AP-1 activity of all-trans retinoic acid in glomerular mesangial cells. *Am J Physiol* **267**: F805–815
Tamimi Y, Bringuier PP, Smit F, van Bokhoven A, Debruyne FM and Schalken JA (1996) p16 mutations/deletions are not frequent events in prostate cancer. *Br J Cancer* **74**: 120–122
Umbas R, Schalken JA, Alders TW, Carter BS, Kathaus HFM, Schaafsma HE, Debruyne FMJ and Issacs WB (1992) Expression of the cellular adhesion molecule E-cadherin is reduced or absent in high-grade prostate cancer. *Cancer Res* **52**: 5104–5109
Umbas R, Isacs WB, Bringuier PP, Scaahsma HA, Kathaus HF, Oosterhof GO, Debruyne FM and Schalken JA (1994) Decreased E-cadherin expression is associated with poor prognosis in patients with prostate cancer. *Cancer Res* **54**: 3929–3933
Wang QM, Jones JB and Studzinski GP (1996) Cyclin-dependent kinase inhibitor p27 as a mediator of the $G_1S$ phase block induced by 1α,25 dihydroxyvitamin $D_3$ in HL60 cells. *Cancer Res* **56**: 264–267
Welsh J (1994) Induction of apoptosis in breast cancer cells in response to vitamin D and antiestrogens. *Biochem Cell Bio* **72**: 537–545
Zhang W, Grasso L, McClain CD, Gambel AM, Cha Y, Travali S, Deisseroth AN and Mercer WE (1995) p53-independent induction of WAF1/CIP1 in human leukaemia cells is correlated with growth arrest accompanying monocyte/macrophage differentiation. *Cancer Res* **55**: 668–674

*© Cancer Research Campaign 1999* 
